# An Accurate Method for Prediction of Protein-Ligand Binding Site on Protein Surface Using SVM and Statistical Depth Function

**DOI:** 10.1155/2013/409658

**Published:** 2013-09-30

**Authors:** Kui Wang, Jianzhao Gao, Shiyi Shen, Jack A. Tuszynski, Jishou Ruan, Gang Hu

**Affiliations:** ^1^College of Mathematical Sciences and LPMC, Nankai University, Tianjin 300071, China; ^2^Division of Experimental Cancer, Cross Cancer Institute, 115660 University Avenue, Edmonton, AB, Canada T6G 2V4

## Abstract

Since proteins carry out their functions through interactions with other molecules, accurately identifying the protein-ligand binding site plays an important role in protein functional annotation and rational drug discovery. In the past two decades, a lot of algorithms were present to predict the protein-ligand binding site. In this paper, we introduce statistical depth function to define negative samples and propose an SVM-based method which integrates sequence and structural information to predict binding site. The results show that the present method performs better than the existent ones. The accuracy, sensitivity, and specificity on training set are 77.55%, 56.15%, and 87.96%, respectively; on the independent test set, the accuracy, sensitivity, and specificity are 80.36%, 53.53%, and 92.38%, respectively.

## 1. Introduction

With the development of the technology to solve the protein structures, the rate of deposition of protein structure in the PDB [1] grows very fast. Unfortunately, there are still a lot of resolved proteins with unknown function. For the proteins always carry out their functions through interactions with other molecules, such as other proteins, peptides, nucleotides, compounds, and so forth, identifying the residues involved in these interactions is an important step towards characterizing protein function. The protein functional sites consist of various types of binding site including protein-ligand binding site, protein-protein binding site, and protein-DNA binding site. Since the ligands (here we refer to small organic compounds as ligands) constitute most of the drugs approved by FDA [2], the prediction of protein-ligand binding site also plays an important role in rational drug discovery. Therefore, we focus on the protein-ligand binding site in this study.

In the past 15 years, many methods were developed to predict protein-ligand binding sites. There are mainly two type of methods, geometry- and energy-based methods. The energy-based methods identify the binding site using model of energetics [3–6], such as PocketFinder [5] and Q-SiteFinder [6]. Most of the algorithms are based on geometry, for the binding sites always locate on the concave surface, which likes a pocket or a cleft. Actually, many methods were proposed to detect the pocket on protein surface using geometric criteria, and the pocket with largest volume is often returned as prediction of binding surface. POCKET [7] and Ligsite [8] map the entire protein structure to 3D grid and cluster the grids with special event, like protein-solvent-protein event or surface-solvent-surface event. Surfnet [9] places empty spheres which separate any two atoms of protein, and cluster these spheres to describe the pocket. PASS [10] uses sphere probes to fill the cavities layer by layer and detect the pocket. CASTp [11] applies the alpha shape theory [12] from computational geometry to detect and measure the pockets. The approaches mentioned above often use pure geometric criteria without additional information like conservation or physic-chemical information. Ligsitecsc [13], ConSurf [14], and ConCavity [15] made a big progress after combining the evolutionary conservation to pocket detections.

Besides methods based on geometry or energy, researchers also developed algorithms based on machine learning model for functional sites prediction. Zhang et al. [16] predict catalytic site using SVM based on sequence information. Ansari and Raghava [17] identify the NAD interacting residues in proteins using SVM. Cheng et al. [18] also use SVMs to predict RNA-binding sites of proteins. Ofran and Rost [19] use neural networks to identify the protein-protein interaction sites. In contrast to the geometry- or energy-based algorithm, the prediction made by machine learning always employed only sequence information and a few structural information like content of secondary structure.

Our study in this paper has two motivations. First, although existent geometry-based methods provided considerable accurate prediction of binding site, improvement could be made by integrating other information, such as evolutionary conservation. Many pure geometry-based methods only return the largest pocket, which is not always true for ligand-binding pocket. Ligsitecsc and ConSurf rerank the pockets by conservation and perform better than previous methods, which rank the pockets by volume. It implies that additional information should be took into account to bind site prediction. On the other hand, machine learning based approaches only focus on sequence information. Here we want to build a method based on comprehensive features which are available and useful for identifying binding site. Not only sequence information but also structural information will be combined together. Second, it is inherently difficult to define negative samples no matter which method employed. It is easy to define positive samples from the interactions of protein-ligand complexes which are experimentally approved. Not all interactions are known, so it is hard to say which residue cannot bind ligands. In this paper, we introduce statistical depth function (details in Section 2) to define the negative samples. The idea is from intuition that binding residues are always locate on concave protein surface and the residues on convex surface are unlikely (not impossible) to bind ligands. 

In this paper we present a novel method base on SVM model integrating both sequence and structural information to predict protein-ligand binding site. To test and validate our method, a benchmark dataset including 373 complexes is built from PDBbind [20]. The validation on the independent test set shows that the accuracy of our method is about 80.36%. For the top 3 pockets provided by Ligsitecsc and CASTp, we rerank them according to our prediction. Then top1 success rate is improved from 41.6% to 75.3% (for Ligsitecsc) and from 61.0% to 77.9% (for CASTp), respectively.

## 2. Material and Methods

### 2.1. Dataset

The PDBbind [20] database provides a collection of experimentally measured binding affinity data exclusively for the biomolecular complexes available in the PDB. We select the “refined set” in PDBbind, which is compiled to provide a high quality set of protein-small ligand complexes. There are 1741 entries in the “refined set” of PDBbind. After removing the redundancy complexes with more than 30% sequence similarity, 373 nonredundant complexes are remained as our dataset. 

We divided these 373 entries into two datasets randomly, one is training set and the other is test set. The training set consists of 296 complexes, and the test set contains 77 complexes. The training set is divided into five subsets (each one has about 60 proteins) randomly for 5-fold cross-validation. The PDB ids of the training set and test set are shown in Support Information STable 1, available online at http://dx.doi.org/10.1155/2013/409658.

### 2.2. Statistical Depth Functions

We employ the statistical depth function to measure the depth of the residue on the protein surface. The statistical depth function gives the residues in the pocket deeper depth values; and for the residues on convex protein surface, it gives them lower depth values. Statistical depth functions assign a point its degree of centrality with respect to a dataset. They are “order statistics” in higher dimension space (≥2). In statistics, statistical depth functions have become increasingly pursued as a useful tool in nonparametric inference for multivariate data. The statistical depth functions have been used to measure the residue depth and analyze the protein structure. There are several statistical depth functions to measure the degree of centrality of a point. In this study, we use half-space depth function to measure the depth of the residues because the concept and the definition of the half-space depth are simple and easy to implement.

*Half-Space Depth.* Tukey [21] introduced the half-space depth to order the high dimensional data. The half depth (HD) of a point *x* in *R* ^*d*^ with respect to a probability measure *P* on *R* ^*d*^ is defined as the minimum probability mass carried by any closed half space containing *x*; that is,

(1) HD(x,P)=inf⁡{P(H):  H  is  a  closed  half  space,  x∈H},                     x∈Rd.

**Figure 1:**
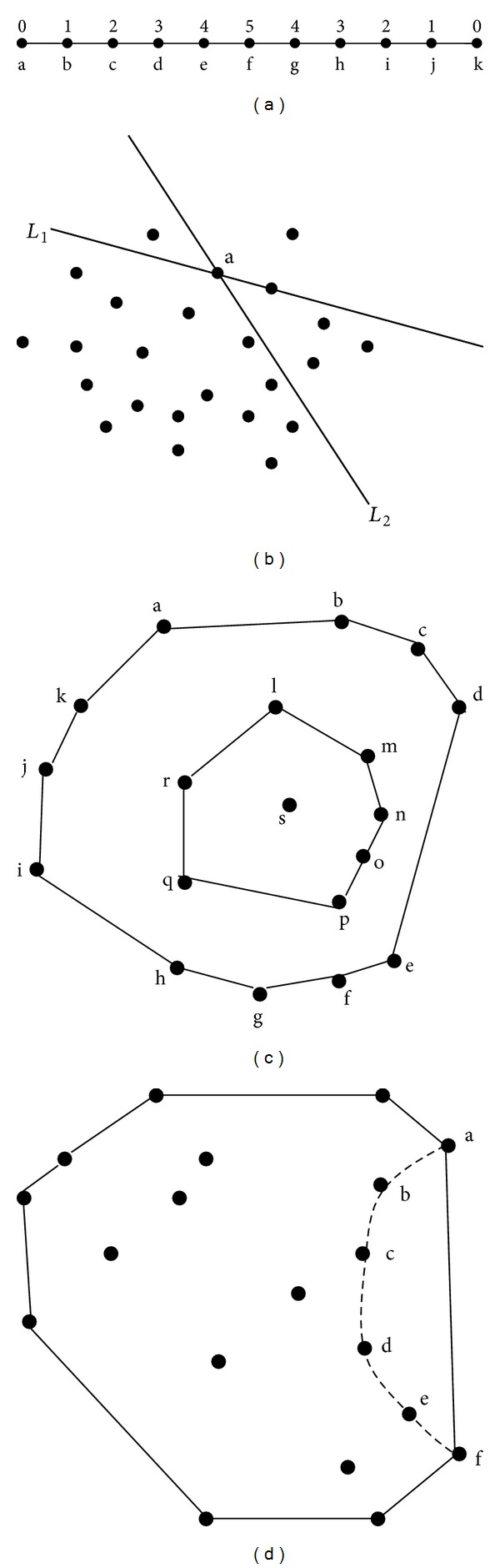
An illustration of the concept of half-space depth for 1- and 2-dimensional datasets.

**Figure 2:**
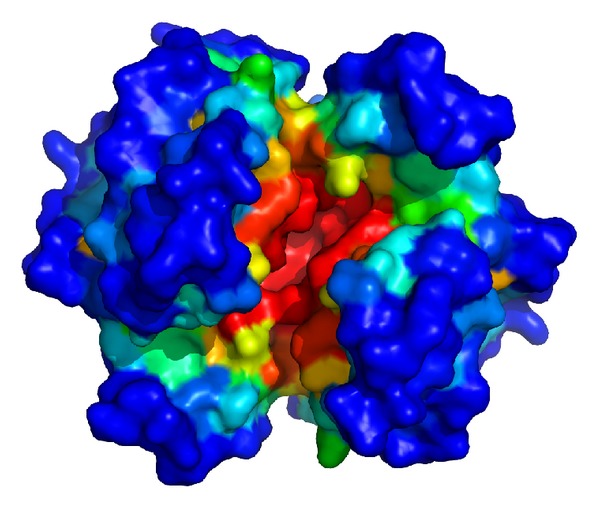
An illustration of the half-space depth function. The protein surface (PDBID: 10 gs) is colored by the statistical depth value of the residues. The color is gradually change from blue to red according to the depth values of the residues. The deepest residues are red, and the convex residues are blue. All the figures of the protein surface are created by Pymol.

For a probability measure *P*, the half-space depth of any point in *R* ^*d*^ with respect to *P* can be defined. For a dataset, such as all the atoms in a protein, we can use the empirical distribution to estimate the probability *P*(*H*). We consider every point in dataset is equiprobable; then *P*(*H*) = ∑*P*(*x*), *x* in *H*, where *P*(*x*) = 1/*n*, *n* is the number of the points in dataset. For simplifying, we define *P*(*x*) = 1; then *P*(*H*) represents the number of the points in *H*. In addition, we define *H* by an open half space. Thus, the depth of the points at the boundary of dataset is zero and the point which has the maximal depth value is the center of the dataset.  Figure 1 shows some examples in 1 and 2 dimensions.  Figure 2 shows the depth function is applied to measure the depth of protein atoms.

*Depth and Relative Accessible Surface Area (RSA)*. According to the definition of half-space depth above, the depth values of buried residues are always greater than zero. And the residues with small depth values locate on the convex of the protein. It is notable that not all the residues with depth values greater than zero are buried, that is, the depth values of the points b, c, d, and e in Figure 1(d). These residues locate in the “pockets” on the protein surface and their RSAs are greater than 0, too. On the other hand, the residues the depth values of which are zero and RSAs of which are greater than zero will locate on the protein surface and will not locate in “pockets” on the protein surface. In addition, using both depth and RSA, the residues in the pockets can be found easily, too. Thus, the residues on the protein surface can be divided into two types according to the RSA and depth value. The first class includes the residues whose RSAs are greater than 0 and depths close to 0, which means these residues locate on the convex of the protein. And the second class includes the residues whose RSAs and depths are both greater than 0, which means the residues in this class locate in pockets on the protein surface.

### 2.3. Sample Selection

Firstly, we introduce some concepts to define the samples. We remove the buried residues and only consider the residues on the protein surface. The residue is considered as being on the surface if its RSA is greater than 10%. For a given residue on the surface, its neighbors are the residues on the surface with distance to the given residue <10 angstroms. We call a residue on the protein surface and its neighbors together a patch. As a result, each residue on protein surface has a patch. A sample just refers to a residue on protein surface or its corresponding patch. In this study, we have three types of samples: positive samples, negative samples, and the others (we call this kind of samples as not positive and not negative samples, NP & NN for short).

For each residue on the surface, if the distance between any nonhydrogen atom of the residue and any atom of the ligand is less than 7 angstroms, this residue is kept as a positive sample candidate; otherwise, the residue is kept as a negative sample candidate. For any positive sample candidate, if more than 30% neighbors of this residue are the positive sample candidates, the sample is considered as a positive sample; otherwise, it is considered as a NP & NN sample.

**Figure 3:**
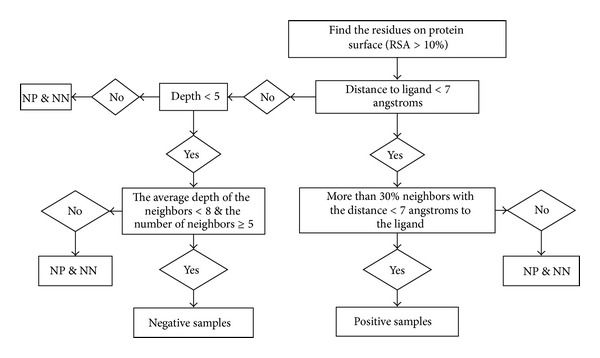
The framework of samples selection.

The negative samples are not easy to define like positive samples. The proteins of complexes might bind some ligands somewhere and we cannot distinguish the potential functional binding residues from the others. We consider the fact that the residues which locate on the protein surface and not in a “pocket” or “cavity” are hard to bind a ligand. So the residues on the convex protein surface are regarded as negative samples in our study. Here we use statistical depth function (which is defined above) to distinguish whether the residue is or is not on the convex surface. Firstly, we remove the residues with half-space depth greater than 5 from the negative sample candidates. The negative sample candidate is defined as negative sample if the average depth value of its neighbors is less than 8 and the number of its neighbors is greater than 5.  Figure 3 shows the framework to select the samples.

Here, the standard of negative samples is a little strict. There are two reasons. One is that we want to make sure our negative samples would not bind to a ligand. The other reason is that the definition can balance the positive samples and negative samples to avoid the training bias. After the samples selection, the radio of positive samples to negative samples is about 1 : 2 in the training set and 1 : 3 in the test set.

### 2.4. Feature Design

There are totally 330 features for each sample. These features include two-aspect information of protein: global information and local information. We explain every feature as follows.

*Global Information*. The global information of the sample includes the length of the protein sequence, the distance to *P* terminal, the distance to *N* terminal, the residue components, and the global secondary structure content. The secondary structures of proteins are calculated by DSSP [22]. We use a four-dimensional binary vector to represent the length and the two distances, which are discredited in four intervals [0, 60), [60, 120), [120, 240), and ≥240. A 20-dimensional vector and a 3-dimensional vector are used for residue components and secondary structure content, respectively. So we have 4∗3 + 20 + 3 = 35 features of global information.

*Local Information.* The local information of the sample comes from two aspects: sequence information and structure information. A sliding window is used for the residue to describe the properties of the neighbors on the protein sequence. Each position in the sliding window includes 30 features. The first 20 features are from position (PSSM-specific scoring matrix) and the 21th is conservation score. The 22th–24th are the 3-dimensional binary vector for the secondary structure of the residue. Then the 25th–29th are positive charge, negative charge, PI value, polarity, and hydrophobicity. The last one is an indicator. If the data do not exist (the side is out of window), all the features are zero and the last feature is assigned 1. We use a window with length 9; thus we have 30∗9 = 270 features about sequence information for the central residue in the sliding window.

The structure information of the sample comes from the patch of this sample. The patch includes the central residue and its neighbors. Because the number of neighbors is not equal, it is impossible to describe every neighbor as the features. As a simplification, we use the minimum, maximum, and the average of the patch properties, which include positive charge, negative charge, PI vale, polarity, hydrophobicity, hydrogen bond tendency, the conservation score, and the secondary structure content. Besides the hydrogen bond tendency, which we use four features to describe (minimum, maximum, average, and the value of the central residue), the secondary structure content needs 3 features to describe and other properties also have three values (minimum, maximum and average of the patch); the total features of local structure information are 4 + 6∗3 + 3 = 25.

Totally, we have 35 + 270 + 25 = 330 features for each sample.

The charges, polarity, and hydrophobicity of amino acid are extracted from AAIndex [23] database. Sarkhel and Desiraju [24] calculated the frequency distribution of hydrogen bonds for amino acids which act as donors or acceptors between proteins and ligands. We use these frequencies as hydrogen bond tendency. The conservation score of the residue is calculated using Shannon Entropy from PSI-blast [25] profile.

### 2.5. Perform the Training and Prediction

**Figure 4:**
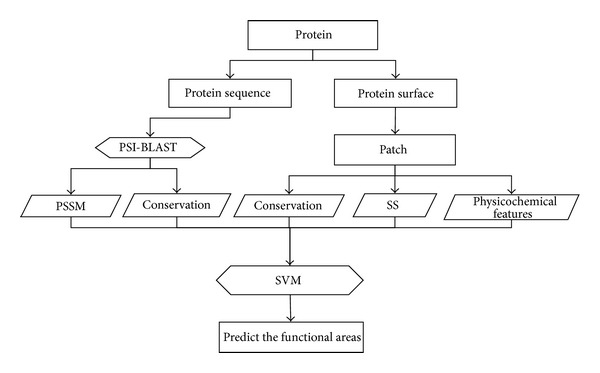
Framework of the proposed prediction system.

As we mentioned in dataset subsection, we divide the dataset into training set and test set. Every feature is normalized to [0,1]. Using Libsvm [26] package and selecting RBF (Radial Basis Function) as the kernel function of SVM, we select 30 proteins from training set to train the SVM parameters using a grid method. Then we train the SVM with the best parameters on training set and predict the functional surfaces on test set. On the training set, we do 5-fold cross-validation to train our model.  Figure 4 shows the framework of the proposed prediction system.

## 3. Results and Discussions

### 3.1. Binding Sites Predictions

Using Libsvm package and selecting RBF as the kernel function of SVM, we train SVM model based on the sequence and structural features mentioned above. Totally, we get 15385 positive samples and 31663 negative samples based on the training set (the ratio between positive samples and negative samples is about 1 : 2). Similarly, we get 3510 positive samples and 12201 negative samples based on the test set (the ratio is about 1 : 3).

The accuracy, precision, sensitivity, specificity, and MCC are used to validate the performance of our method. Then these five indices of training set and the test set are shown in Table 1. We do 5-fold cross-validation to avoid overfitting problem. And the accuracy of the train set is shown similar to the accuracy of the test set. It can also be observed in the case of precision, sensitivity, specificity, and MCC. 

CASTp returns the exact binding residues and the residues forming the pocket mouth. We can compare the binding prediction between our method and CASTp directly, which is shown in Table 1. Our method clearly outperforms CASTp.

**Figure 5:**
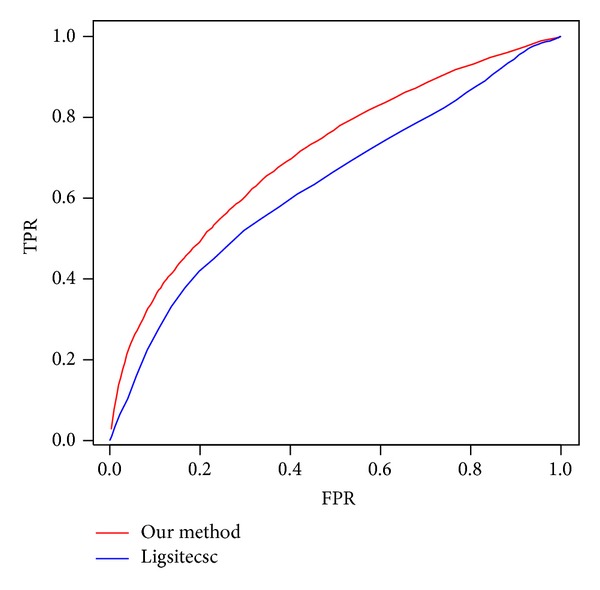
The ROC curves of our method and Ligsitecsc. The blue line is ROC curve of Ligsitecsc, and the red line is of our method.

Unfortunately different approaches always return the predictions in different ways. For example, Ligsitecsc only returns the geometry center coordinates of the pocket. To compare our method with other algorithms, we have to evaluate them using the same standards. Therefore, we define the residues close enough to the pocket center as the binding site/binding residues returned by Ligsitecsc. The distance threshold to pocket center ranges from 1 A to 75 A, because the binding residues will not increase after the threshold greater than 75 A. We can also obtain the ROC curve of the Ligsitecsc (Figure 5) by this way. Since the SVM model returns a real value range from 0 to 1, the ROC curve of our method is also shown in Figure 5. From the comparison of ROC curves, our method performs much better than Ligsitecsc. The AUC value of our method is 0.71 greater than 0.63 which is the AUC vale of Ligsitecsc.

### 3.2. Rerank the Pockets

Although our method can identify the binding sites more accurately than CASTp and Ligsitecsc, we cannot provide a pocket-level comparison. Unlike CASTp and Ligsitecsc, we do not detect pocket. However, we can also apply our model in the postprocess of geometry-based models to improve the rank of the pockets. The motivation is that using conservation score not pocket volume has made a big progress in the search of pocket. 

We focus on the top 3 pockets obtained from CASTp and Ligsitecsc. For Ligsitecsc, it only gives three pockets per protein and ranks them using the conservation score. The top 1 pocket in Ligsitecsc means the most conservative pocket in three of them. The CASTp provides more pockets than Ligsitecsc, but the pockets are not ranked and many of them are too small to bind ligand. We rank them using volume and select the three largest ones as the top 3 pockets returned by CASTp. The pocket from prediction is considered as a functional or binding pocket when the distance between the pocket geometry center and any atom of ligands is less than 8 A. We define the top *n* (*n* = 1, 2,3) success rates as the number of functional pockets in top n divided by the number of proteins. On our test set, we have 77 proteins and 231 prediction pockets in total.

Our method is combined to pocket detection algorithm by a simple way. For each pocket from prediction, we only count the residues which are predicted as binding sites by our SVM model. Then this number is used to rank the pocket. The result is shown in Table 2. Top 3 success rates among the four methods are the same according to the definition, but the top 1 success rates are totally different. The methods combined with our model gain a much improvement from the CASTp or Ligsitecsc. It implies our method included the complementary information for CASTp and Ligsitecsc. And the model can be applied in other geometry-based pocket detection methods. 

## 4. Conclusion

In this paper, we introduce statistical depth function to define the negative sample of the protein-ligand binding site. The further analysis shows negative sample defined by this way is reasonable and helpful for the model training (shown in Support Information Sections 2–4). Then we propose an SVM model including sequence and structural information; the results show the method significantly outperforms the existing methods based on pure geometry or only combining evolutionary conservation. Our method can also provide the complementary information for geometry-based methods such as CASTp and Ligsitecsc in the postprocess.

## Supplementary Material

The Supplementary Material includes four sections. The pdb ids of dataset used in paper are provided in the first section. The statistics of physiochemical properties in different samples is shown in the second section. The impact of NP&&NN samples is further discussed in the third section. In the fourth section, we evaluate the performance if the depth is introduced to the model of prediction.

## Figures and Tables

**Table 1:** The values of five indices on the training set and test set for well-trained SVM.

Dataset	Accuracy	Precision	Sensitivity	Specificity	MCC
Train set	77.55%	69.38%	56.15%	87.96%	**49.68%**
Test set (our method)	**80.36%**	**75.88%**	**53.53%**	**92.38%**	**50.85%**
Test set (CASTp)	**56.49%**	**20.81%**	**41.67%**	**60.21%**	**2.0%**

**Table 2:** Top *n* success rates for test set.

Methods	Top 1	Top 2	Top 3
Ligsitecsc	40.3% (31/77)	71.4% (55/77)	83.1% (64/77)
Ligsite + our method	**75.3% (58/77)**	**81.8% (63/77)**	**83.1% (64/77)**
CAStp	60.0% (47/77)	88.3% (68/77)	92.2% (71/77)
CAStp + our method	**77.9% (60/77)**	**90.9% (70/77)**	**92.2% (71/77)**
