# Supplementary material for: An Accurate Method for Prediction of Protein-Ligand Binding Site on Protein Surface Using SVM and Statistical Depth Function
Source: Biomed Res Int. 2013 Sep 30;2013:409658. doi: 10.1155/2013/409658 (PMC3806129; doi:10.1155/2013/409658)
Supplement: Supplementary file 1 — The Supplementary Material includes four sections. The pdb ids of dataset used in paper are provided in the first section. The statistics of physiochemical properties in different samples is shown in the second section. The impact of NP&&NN samples is further discussed in the third section. In the fourth section, we evaluate the performance if the depth is introduced to the model of prediction. [file 409658.f1.doc]

**An accurate method for prediction of protein-ligand binding site on protein surface using SVM and statistical depth function**

**Support Information**

1. **The PDB ids of training set and test set**

| **Training Set** |
| --- |
| 2q88 1m13 1kf0 3cm2 1ua4 1a99 2gz2 1vkj 2ima 2jbj 1wei 1upf 1m1b 1a4r 1yqy 2v2h 1sw1 2jiw 1gcz 2oxy 5yas 1m0n 2g8r 1tok 1urg 2j4k 3cpj 2h6t 1wm1 2br6 1k1y 1r6n 1jzs 1r4w 2vw5 2vt3 1nki 2qv7 2cbj 1h0a 2o8h 1hlk 3pce 1a08 1t7d 2p09 2pqc 2aj8 2mas 1olx 1m6p 3bf1 1a8i 2evl 1jt1 1r0x 1moq 3eqr 1j36 2h6q 3e5u 1y8o 1q95 2zcs 1tuf 2bvd 1gyx 2pnc 3d7k 2qbu 1s5z 1e5a 1dy4 2fqt 1eix 2zif 1ogz 2jkp 2v95 1ajp 1dqx 1o8b 2i3h 1duv 1lrt 2b4l 2gsu 1uwf 2ihj 2qmj 1szd 2qfu 2hhn 1alw 1y4z 2hqu 2gvj 3b4p 1br6 1d2e 1m2x 1q5k 1k9s 1nw7 2a5b 1gai 1qca 1xk9 10gs 1oba 1bai 2jdy 2v54 1s63 1we2 3c2r 2isv 1t5f 1j4r 1dfo 1lox 1ikt 1hi5 2gyi 2hzl 1drv 2toh 1ws1 1qq9 1ax0 3b2q 1r9l 6std 1bzy 2cht 2pu2 1l6m 2afw 1nc1 1i5r 2hf8 1pdz 2pfy 1q54 1rql 2fqy 2bt9 2gst 1r1j 1ogd 1h1p 2gl0 1qi0 2zm3 1jwt 1zky 2q8z 3eeb 1n4k 1n1t 2i2c 2fwp 2ews 2r75 1c3x 3ckb 1qiw 1fzq 2hjb 3coz 1m5w 2i80 1jqy 1rd4 1pbq 1vyg 1lyb 2bfr 3bfu 2hrm 1g9r 1i00 1oko 1njs 3f8f 1dzk 1l83 1nje 2e94 3jdw 2qm7 1uho 1k27 2vfk 1icj 2aov 1aj6 2h5a 1rpj 1swr 1ofz 1q1g 2qm9 2epn 1efy 1tkb 1lrh 2vuk 2i4z 1ez9 2pql 1kc7 2c80 3bxh 1uou 1xk5 1p19 1dqn 1v1j 1grp 1h6h 1e3v 1zhy 3cd7 1b3l 2doo 2amt 2j78 1jcx 3ebl 1sqn 1q91 1elr 1ew8 1pfu 3cj5 1jlr 6rnt 2hxm 1koj 1mai 2g72 5tmp 2fgi 2gvv 2e5y 2r0h 2o1c 2rio 2v8w 3c2u 2aac 1wcq 1ws4 2qry 2byr 1ch8 2pwd 2ha3 1oar 3e8r 1atl 2qpu 1n4h 2qt5 1yvm 1ydk 2qrl 1wvc 1x8j 1qji 2za0 1f4f 1pzp 2csn 1jq8 2glp 1ro6 3b5j 3cke 1ow4 1m83 1gwv 2z8e 1ado 1pkx 1hp0 1fh7 1kjr 1n2v 2fxv 2rk8 3b3c 1m48 1lbf 1ec9 |
| **Test Set** |
| 1n0s 1n51 3cd5 1a94 1rdt 3brn 1xge 2rcn 2vj8 2yz3 1jak 3e5a 1fiv 2d1o 3d52 1nli 2qx0 1u1w 1qan 1kmy 1tmn 1obx 2sim 1uj5 2oyk 1n8v 2p4s 2g79 2bet 1pa9 1kyv 1fcy 3d0e 2fu8 1nf8 1kdk 1pvn 1ork 1e6q 1byk 1ui0 2f5t 1s89 1qy2 3clp 1pgp 1y3p 2vyt 1e2k 2ogy 1vjj 3czv 1ugp 1b55 1wur 2d0k 1hee 1z4o 1gz3 1fao 2itk 1xgi 2oi2 1hyo 1ppi 2ewb 1yon 1ro7 1wn6 1ctt 2rcb 1bq4 1nu3 1m7y 2am4 2q6f 2fxu |

**STable 1. The PDB ids of training set and test set**

There are 296 complexes and 77 complexes in training set and test set respectively.

1. **Descriptive statistics of binding residues.**

For comparison with negative samples, we analyzed the depth value distribution on positive samples. This definition of negative samples is based on the observation that most of the ligands bind to pockets or cavities on protein surface which will have a large depth value. To validate this observation, we calculated all the depth values of the positive samples on our dataset. STable 2 shows the means, standard deviation, max, min of the depth values in positive, negative and NP&NN samples.

STable 2. The descriptive statistics of the depth values of residues.

|  | Min. | Mean | Max. | Std |
| --- | --- | --- | --- | --- |
| Positive | 0.25 | 51.1 | 690.6 | 99.08 |
| Negative | 0 | 3.59 | 7.92 | 1.30 |
| NP&NN | 0 | 49.5 | 822 | 91.83 |
| All | 0 | 36.18 | 822 | 82.39 |

From the STable 2, we found that most of positive samples have a greater depth values. That means the ligands prefer to bind the patches with larger depth values on protein surface. In the other words, it is reasonable for us to define the convex residues as the negative samples for SVM training. The residues which are NP&&NN samples have the similar but still less depth values than positive samples. These residues probably can bind some ligands.

STable 3. The descriptive statistics of the polarity of residues.

|  | Min. | Mean | Max. | Std |
| --- | --- | --- | --- | --- |
| Positive | -1.05 | -0.12 | 1.26 | 0.80 |
| Negative | -1.01 | -0.22 | 0.84 | 0.70 |
| NP&NN | -1.09 | -0.19 | 1.25 | 0.78 |
| Surface residues | -1.09 | -0.19 | 1.26 | 0.76 |

Besides depth, the physiochemical properties of residues also affect their binding ability. We compare the physiochemical properties of the residues in different samples and the residues on surface. STable 3 and STable 4 show the statistics of the polarity of the single residues and the patch. From these 2 tables, we found that the polarity of the positive samples and negative samples have a great difference. The positive samples have much more polar residues than negative samples, which suggest that the ligand prefer to bind the polar residues. For NP&NN samples, because some of them might bind ligands, as expect, the mean polarity values of NP&NN samples are less than positive samples but greater than negative samples .

STable 4. The descriptive statistics of the polarity of patch.

|  | Min. | Mean | Max. | Std |
| --- | --- | --- | --- | --- |
| Positive | -1.180 | -0.09255 | 1.360 | 0.26 |
| Negative | -1.180 | -0.302 | 1.360 | 0.24 |
| NP&NN | -1.180 | -0.1792 | 1.360 | 0.24 |
| Surface residues | -1.180 | -0.2032 | 1.360 | 0.25 |

In fact, not only on the polarity, we get the similar results on other three features. SFigure 1 shows the means of these four features, polarity, hydrophobicity, hydrogen bond tendency and conservation. In these four features, the means of the NP&NN samples is always between the means of positive samples and the means of negative samples. This means that NP&NN samples may contain the binding residues which we have not found yet. These results suggest that our definitions for positive and negative samples selection is reasonable.


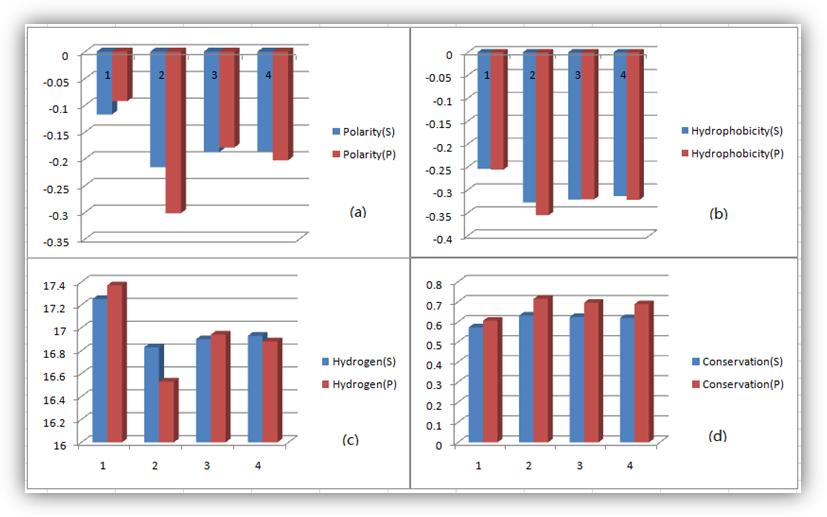


SFigure 1. The means of features. (a) Polarity (b) Hydrophobicity (c) Hydrogen bond tendency (d) Conservation. 1,2,3 and 4 in figures represent positive samples, negative samples, NP&NN samples and all samples. The blue bar represents the means of single residues and the red bar represents the means of patch.

Because our samples are all the residues on the protein surface, the hydrophilic residues are majority of all samples. The means of the hydrophobicity of these four types of samples are all less than zero (SFigure 1b). However, the mean of hydrophobicity of positive samples is greater than other samples and the mean of negative samples is lowest. This suggests that the binding residues are more hydrophobic than the other residues on protein surface. SFigure 1c shows the binding residues are more likely to be hydrogen bond donors or hydrogen bond acceptors. Many studies suggest the binding residues are more conserved than the other residues. SFigure 1d shows the means of the conservation on four classes. Obviously, the binding sites (positive samples) are more conserved than other areas. This result is consistent with previous findings.

Although the results above are similar on singe residues (blue bars) and on patch (red bars), there are a little difference from these 4 features. For hydrophobicity and conservation, there is little difference between single residues and patches. But for polarity and hydrogen bond tendency, the difference between single residues and patches is big (SFigure 1). The means of the patches vary wider than the means of single residues with these 4 classes. This result implies that the polarity and hydrogen bond tendency of the neighbor residues might have effect on ligand-residue binding. This is a reason we use patch to define the positive and negative samples and the features include not only the properties of the single residue but also the properties of the neighbor residues.

We continue to consider the difference based on the other features. For a set with N residues, its secondary structure content is a 3-dimentional vector (h, e, c), in which, h is the rate of all residues of this set whose secondary structure is helix; e is the rate of all residues of this set whose secondary structure is strand; and c is the rate of all residues of this set whose secondary structure is coil. For example, in our dataset, the secondary structure content of the binding residues is (0.29, 0.18, 0.53), while the secondary structure content of the negative samples is (0.36, 0.10, 0.54). Then the difference between two the secondary structure contents is significant.

**3. Impact of NP&&NN samples** Someone may argue that there are no pre-defined positive samples and negative samples for us to predict in the real world, for these two samples are not cover all the patches on the protein surface. What about those patches which belong to neither positive nor negative samples? We have defined them as NP&&NN samples before. To investigate the NP&&NN samples, we define two indices:

Tx rate = Tx/(Tx+Fx)

Fx rate = Fx/(Tx+Fx)

where TX refers the number of NP&&NN predicted as positive and FX refers the number of NP&&NN predicted as negative. Tx rate and Fx rate refer the rate predicted as positive and negative in NP&&NN samples respectively.

Because there is no evident to prove the NP&&NN can or can not bind ligand, it’s possible that some NP&&NN can bind ligand which is unknown now. But the probability of NP&&NN to bind ligand is not easy to estimate. The Tx rate is apparently correlated with this probability. Because the larger the protein size is, the more samples are extracted, the indices calculated over all the samples may introduce bias. We investigate the prediction over each protein.

The following figure shows the histogram of tp rate (sensitivity) and tx rate**.**

| 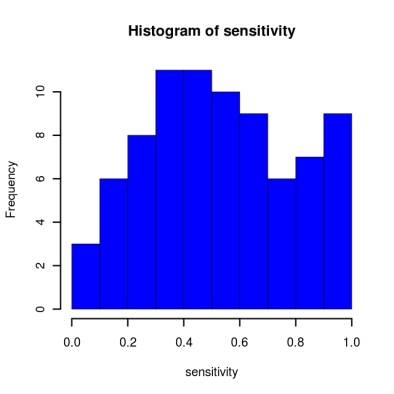(a) | 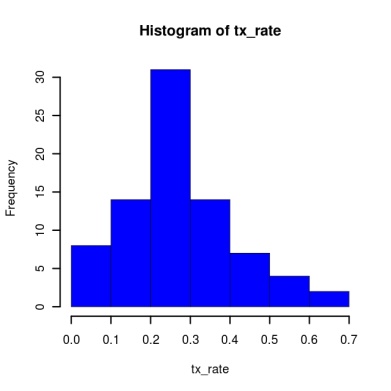(b) |
| --- | --- |

SFigure 2. The histogram of tp rate (sensitivity) and tx rate. (a) is histogram of tp rate, which shows most of the protein have a tp rate more then 0.5. (b) is histogram of tx rate, which shows most of NP&&NN samples in different proteins are predicted as positive less than 0.3.

From SFigure 2, it is clear the most of proteins have the sensitivity more than 50%. Some predictions achieve nearly 100%. This implies ligand binding site of some proteins are much easier to predict.

For the NP&NN samples, we show the histogram of tx rate in SFigure 2 (b).

The most of NN&NN samples are predicted as negative, it is easy to see that the tx rate is less than tp rate on average. This result is reasonable. The first reason is that the unknown sites have less probability to bind ligand. The fact that tx rate and tp rate are not correlated with each other implies the NP&&NN samples are predicted as positive not by randomly but by its intrinsic properties.

**4. Impact of depth:** The statistical depth is used to identify the negative samples in our methods. And the relationship between the depth and the positive samples are illustrated above. The conclusion is most of the binding surface has greater depth value. This fact induces us to investigate the impact of depth on the prediction. If the depth is induced to the model of prediction, can we achieve higher accuracy? The depth index is considered as a threshold, which will remove the predicted positive pixel with smaller depth value. For the depth value is related with the protein size, we use depth rate, which is define as below:

Depth rate=depth / protein size

We investigate the depth index on the test set. For a given depth rate threshold, the new predicted positive should be predicted as positive originally and have a depth rate more than the threshold. When the depth rate threshold increase, this operation will decrease Tp/Fp/Tx and increase Tn/Fn/Fx, which is easy to deduce that the specificity(Tn/(Tn+Fp)) will increase and the sensitivity(Tp/(Tp+Fn)) and Tx rate(Tx/(Tx+Fx)) will decrease. For the depth rate threshold increase from 0 to 0.1, the SFigure 3 below shows that the sensitivity and Tx rate decrease linearly, and the specificity increases exponentially on the contrary. It is not trivial that the precision increases exponentially, which implies the Fp decreases much faster than Tp. This illustrate that the area with large depth value are tendency to bind ligand.


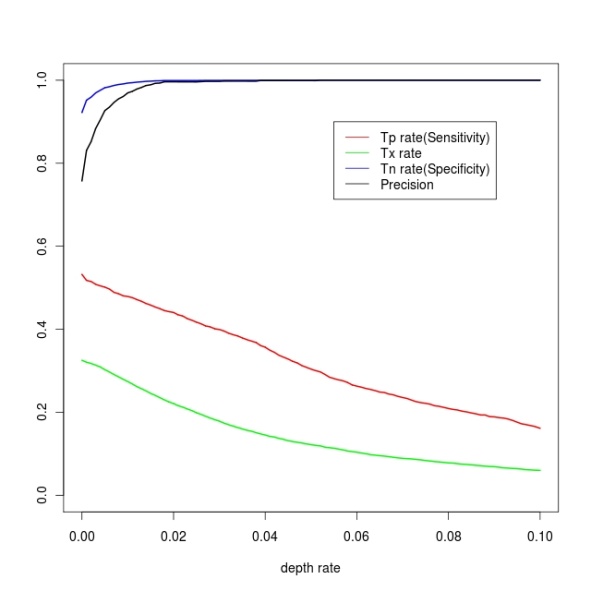


SFigure 3. Tp/Tx/Tn rate and precision curve according to depth rate from 0 to 0.1

However, it is not true that the larger depth rate threshold is, the better accuracy is. The accuracy and mcc indices are interesting and different from other indices, which are showed in SFigure 4. The curves are not monotonous monotone increasing or decreasing. The maximum accuracy and mcc are 83.20%, 60.53% respectively, when the depth rate is 0.011(STable 5). Furthermore the average depth value is nearly 5.79, which is between the 3^rd^ Qu value of depth on negative samples and the 1^st^ Qu value of depth on positive samples. Except the sensitivity decrease 6%, other indices in the table make a significantly improvement by using the depth rate threshold. This implies the depth index do a really useful to predict the binding surface with large depth.

STable 5 The prediction result on test set after using the depth rate threshold 0.011

| Accuracy | 83.20% |
| --- | --- |
| Precision | 96.94% |
| Sensitivity | 47.89% |
| Specificity | 99.31% |
| MCC | 60.53% |

| 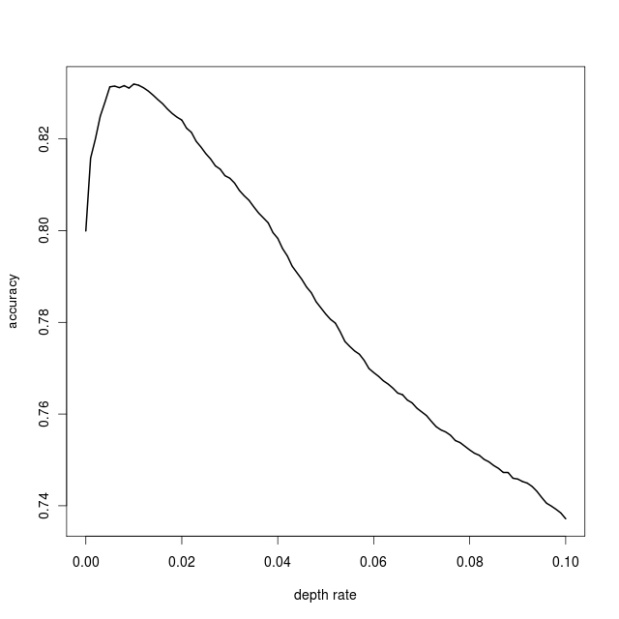  (a) | 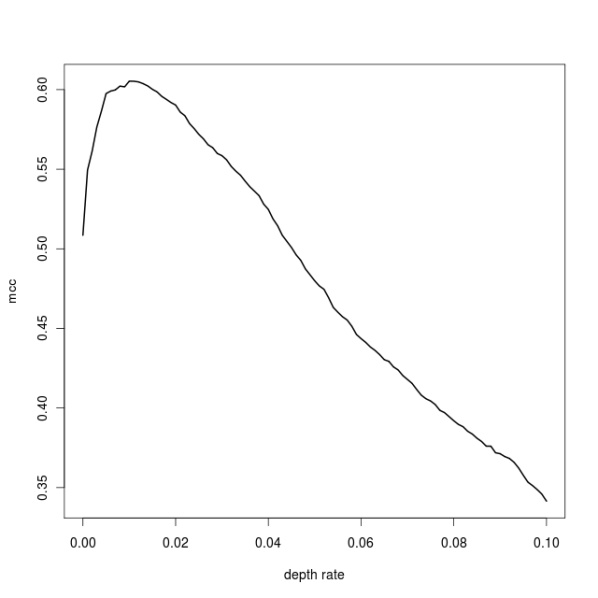  (b) |
| --- | --- |

SFigure 4 (a) The accuracy curve according to depth rate from 0 to 0.1. (b) The mcc curve according to depth rate from 0 to 0.1.
